# Concentrations and content of mercury in bark, wood, and leaves in hardwoods and conifers in four forested sites in the northeastern USA

**DOI:** 10.1371/journal.pone.0196293

**Published:** 2018-04-23

**Authors:** Yang Yang, Ruth D. Yanai, Charles T. Driscoll, Mario Montesdeoca, Kevin T. Smith

**Affiliations:** 1 Department of Forest and Natural Resources Management, State University of New York College of Environmental Science and Forestry, Syracuse, NY, United States of America; 2 Department of Civil and Environmental Engineering, Syracuse University, Syracuse, NY, United States of America; 3 USDA Forest Service, Northern Research Station, Durham, NH, United States of America; Chinese Academy of Forestry, CHINA

## Abstract

Mercury (Hg) is deposited from the atmosphere to remote areas such as forests, but the amount of Hg in trees is not well known. To determine the importance of Hg in trees, we analyzed foliage, bark and bole wood of eight tree species at four sites in the northeastern USA (Huntington Forest, NY; Sleepers River, VT; Hubbard Brook, NH; Bear Brook, ME). Foliar concentrations of Hg averaged 16.3 ng g^-1^ among the hardwood species, which was significantly lower than values in conifers, which averaged 28.6 ng g^-1^ (*p* < 0.001). Similarly, bark concentrations of Hg were lower (*p* < 0.001) in hardwoods (7.7 ng g^-1^) than conifers (22.5 ng g^-1^). For wood, concentrations of Hg were higher in yellow birch (2.1–2.8 ng g^-1^) and white pine (2.3 ng g^-1^) than in the other species, which averaged 1.4 ng g^-1^ (*p* < 0.0001). Sites differed significantly in Hg concentrations of foliage and bark (*p* = 0.02), which are directly exposed to the atmosphere, but the concentration of Hg in wood depended more on species (*p* < 0.001) than site (*p* = 0.60). The Hg contents of tree tissues in hardwood stands, estimated from modeled biomass and measured concentrations at each site, were higher in bark (mean of 0.10 g ha^-1^) and wood (0.16 g ha^-1^) than in foliage (0.06 g ha^-1^). In conifer stands, because foliar concentrations were higher, the foliar pool tended to be more important. Quantifying Hg in tree tissues is essential to understanding the pools and fluxes of Hg in forest ecosystems.

## Introduction

Forests are important receptors of atmospheric mercury (Hg) deposition mainly because of the large surface area of foliage that collects Hg [1]. Trees are second only to soils as the dominant Hg pool in forest ecosystems. Trees contribute Hg to the forest floor via litterfall [2,3] and throughfall [4, 5]. Trees not only absorb Hg through phloem via foliar stomata but also take up Hg through xylem sap via roots [6]. Tree foliage can also re-emit Hg back to the atmosphere via transpiration [7]. Thus, trees mediate bi-directional transport of Hg between the atmosphere and soils. Research on Hg in foliage improves understanding of Hg bioaccumulation in the terrestrial food chain as tree foliage is an important food source for herbivores [8, 9]. Understanding the content of Hg in tree tissues would also help in the prediction of re-emission of Hg associated with losses of aboveground carbon pools [10]; biomass burning is a potentially important but poorly characterized source of Hg emissions [11].

Studies have been conducted to determine Hg concentrations in various tree tissues including foliage [3, 12–14], leaf litter [15–17], bark [18, 19] and roots [20]. Concentrations of Hg in wood are much lower than in other tissues. Studies of wood Hg have examined historical Hg deposition via the accumulation of Hg in tree rings using conifer species [21–24]. Mercury in wood of hardwood species has also been reported but is often below detection limits [25] especially for maple (*Acer* spp.) [23, 26]. However, because wood is the largest component of forest biomass, it can represent a larger Hg pool than foliage [27] and thus wood can be as important as foliage and bark to Hg budgets in forests in spite of its low concentrations.

Differences in tree Hg concentrations with geographic location have been explained by variation in atmospheric Hg deposition. For example, Hg concentrations in foliage varied across sites within the Adirondack Park in New York due to differences in atmospheric Hg deposition [28]; across the continental United States, concentrations varied with annual precipitation [25], which is correlated with Hg deposition. Tree species within a site can differ in Hg concentration [17, 27], presumably due to different rates of absorption through stomata or uptake via roots. Lack of information on Hg concentrations from multiple species and tissue types across a geographic gradient makes it difficult to evaluate the relative importance of species differences in Hg accumulation rates versus differential exposure to atmospheric deposition. For example, the previous study that compared 14 sites across North America rarely observed the same species at multiple sites [25].

The first objective of this study was to determine the concentrations of Hg in foliage, bark, and wood across four study sites for eight tree species, five of which occur at more than two sites. We compared the importance of site and species in controlling tree Hg concentrations to indicate the relative importance of Hg exposure and the properties of tree species. We expected Hg concentrations in foliage and bark to show more influence of location as they are more exposed to the atmosphere to than is wood. Concentrations of Hg in wood were expected to be more consistent across sites because of the limited translocation of Hg to wood. Our second objective was to compare Hg pools among tissue types and between conifer and hardwood stands. We expected woody tissues such as bark and wood to contain more Hg than foliage on a land-area basis because of their greater mass.

## Materials and methods

### Site descriptions

**Fig 1:**
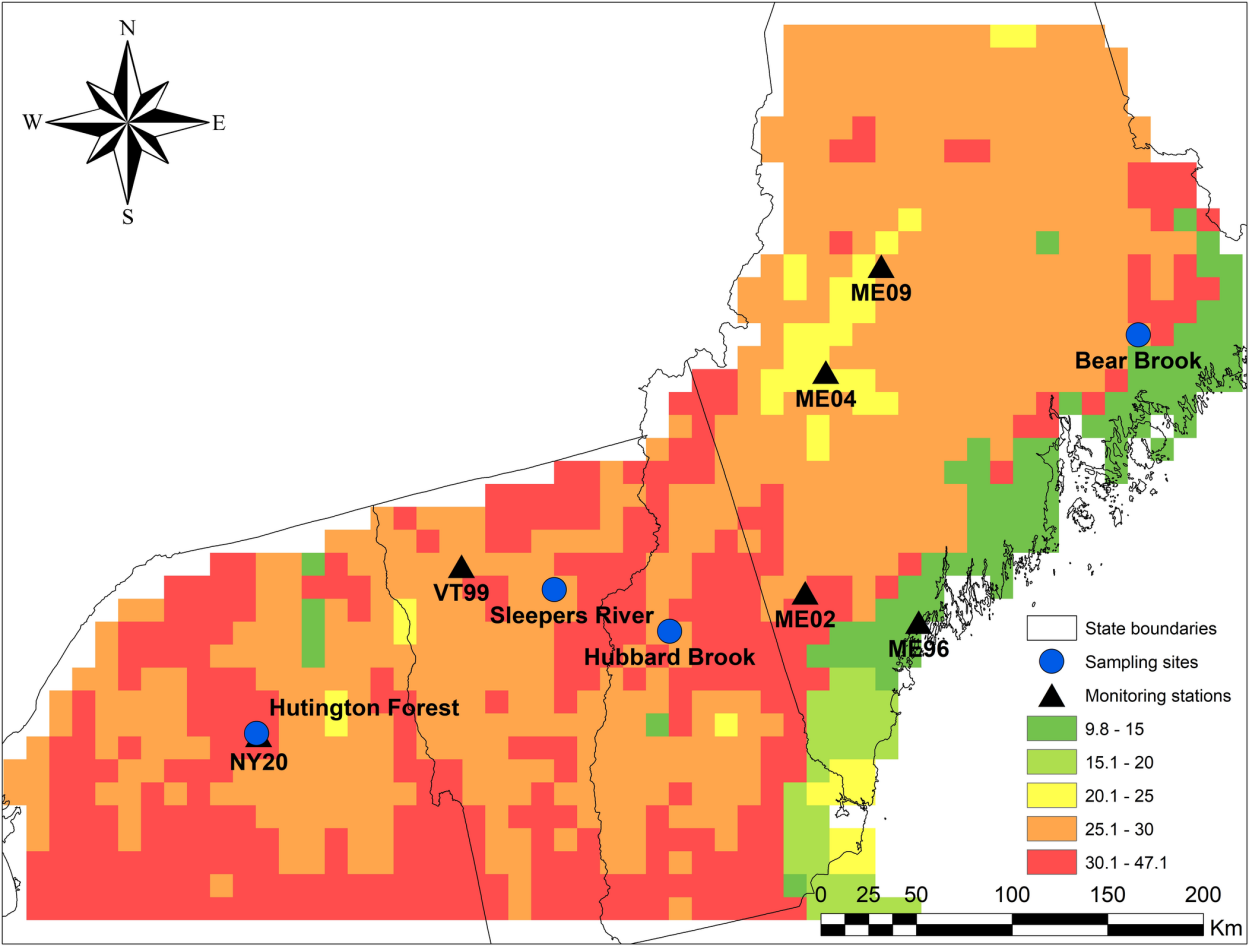
Sampling locations in this study (circles) and monitoring stations (triangles) in northeastern USA. The base map shows the sum of dry and wet Hg deposition modeled by Yu et al. (2014).

Four forested sites in the northeastern USA, each with both hardwood and conifer stands, were selected for sample collection (Table 1), based on the availability of tree inventory data and previous Hg studies of throughfall, litter, soil and streams [17, 29]. Permissions to conduct field studies were provided by employees of the State University of New York College of Environmental Science and Forestry for the Huntington Wildlife Forest in NY, employees of the US Forest Service for the Hubbard Brook Experimental Forest in NH, employees of the US Geological Survey for Sleepers River Research Watershed in VT, and employees of the University of Maine for Bear Brook Watershed in Maine. At the Huntington Wildlife Forest in New York [30], the Hubbard Brook Experimental Forest in New Hampshire [31], and Bear Brook Watershed in Maine [32], soils are dominantly well-drained Haplorthods developed in glacial drift. At Sleepers River Research Watershed VT, the conifer stand is on similar Spodosols, but the hardwood stand is on richer Inceptisols with a carbonate influence [33]. All our study sites were affected by anthropogenic Hg emission [1] from powerplants in the Midwest and urban centers of the Northeast [34, 35]. Total atmospheric Hg deposition was estimated at 32 μg m^-2^ yr^-1^ at Huntington Forest in the Adirondacks, 29 μg m^-2^ yr^-1^ at Sleepers River and Hubbard Brook in central New England, and 27 μg m^-2^ yr^-1^ at Bear Brook Watershed in Maine (Fig 1), using data from Yu et al. [36]. In that study, pools of Hg in soil organic horizons averaged 4.6 mg m^-2^ in the Adirondacks, 3.5 mg mg^-2^ in central New England, and 2.7 mg m^-2^ in Maine [36]. Mineral soil Hg pools averaged 18, 18, and 19 mg m^-2^ in these three regions, such that all three regions had a total soil Hg pool of 22 mg m^-2^ [36].

**Table 1:** Four sites in the northeastern USA were used in this study.

Study location	Stands	Sampled species^a^	Annual mean temperature (°C)	Annual mean precipitation (cm)	Latitude (N)	Longitude (W)	Elevation (m asl)
Huntington Forest, NY	Hardwood	BE, YB, SM	5.0	105	43°59'2.3''	74°14'1.1''	530
	Conifer	WP, BF			43°58'25''	74°13'26''	508
Sleepers River, VT	Hardwood	WA, YB, SM	6.0	110	44°29'54''	72°09'33''	540
	Conifer	RS, BF			44°30'46''	72°10'47''	670
Hubbard Brook, NH	Hardwood	BE, YB, SM	5.7	140	43°56'13''	71°74'20''	500
	Conifer	RS, BF			43°56'24''	71°44'24''	780
Bear Brook, ME	Hardwood	BE, YB, RM	5.2	132	44°51'36''	68°6'17''	430
	Conifer	RS			44°51'42''	68°6'12''	446

^a^ tree species included American beech (BE), yellow birch (YB), red maple (RM), sugar maple (SM), red spruce (RS), white ash (WA), white pine (WP) and balsam fir (BF).

### Field sampling

Dominant hardwood and conifer species were sampled in each stand, including American beech (*Fagus grandifolia* Ehrh.), white ash (*Fraxinus americana* L.), yellow birch (*Betula alleghaniensis* Britt.), sugar maple (*Acer saccharum* Marsh.), red maple (*Acer rubrum* L.), red spruce (*Picea rubens* Sarg.), balsam fir (*Abies balsamea* (L.) Mill.) and white pine (*Pinus strobus* L.). Five of these species were sampled from at least three sites (Table 1). Nine individual trees of each species in dominant canopy positions were sampled from August 7^th^ to 13^th^ 2015. For American beech, we preferentially selected trees not severely affected by beech bark disease.

For each tree, bark without visible lichen was collected from the stem 1.3 m above the ground with a chisel and hammer. Two cores were taken from the outer wood to the pith from each tree at 1 m above the ground using a Pressler increment borer 5 mm in diameter. The two cores were combined to form a single sample for each tree.

Sun-exposed leaves or needles without evidence of herbivory or pathogens were collected from the upper canopy position using a shotgun at Sleepers River and Hubbard Brook. At Huntington Forest and Bear Brook, foliage samples were collected using ladders and pole pruners. For conifers, needles of all age classes were pooled from a lateral branch to form a single foliage sample for each tree. To avoid contamination, samples were collected wearing nitrile gloves and tools were rinsed with methanol between samples. Samples were stored in doubled Ziploc bags on ice in the field and kept frozen in the laboratory until further analysis.

### Laboratory analysis

Samples were cleaned using DI water and freeze-dried to constant mass at -80°C and 7 Pa, using FreeZone Plus 6 (Labconco, Kansas City, MO). Dried samples were ground into homogenized particles using a Freeze Mill (Metuchen, NJ). For each type of tissue and species, samples were composited in groups of three trees prior to Hg analysis. This approach gives a more accurate estimate of the population mean than a sample of three trees, for the same analytical effort.

Composited samples were analyzed for Hg concentration using thermal decomposition, catalytic conversion, amalgamation, and atomic absorption spectrophotometry [37], using a Milestone DMA 80 direct Hg analyzer (Shelton, CT) [38]. For each sample, two replicate samples of ~100 mg of tissue were weighed into nickel boats and auto-loaded into the instrument. Aluminum oxide was added to each tissue sample to ensure that the samples were fully burned. The reported Hg concentration is the average of the two replicate samples.

### Quality control

Before running tissue samples, we analyzed a batch of blanks, primers (NIST DORM-2, dogfish muscle, ~50 mg, 410 ± 41 ng g^-1^), calibration verification samples (NIST 2976 mussel tissue, ~15 mg, 61 ± 6 ng g^-1^), two quality control samples (NIST 1515 apple leaves, ~5 mg, 44 ± 4 ng g^-1^) and one method blank with aluminum oxide. We did not proceed with sample analysis unless the Hg recovery values of NIST reference materials were within 10% of the certified values. After every 10 samples, we ran a calibration verification (NIST 2976), and a calibration blank and a matrix spike. The matrix spike was one actual tissue sample spiked with the standard reference material (NIST 2976). The average Hg recovery was 99% (n = 32, rsd = 8%) of NIST 2976, 100% (n = 16, rsd = 5%) of DORM-2, 100% (n = 8, rsd = 5.7%) of NIST 1515 and 107% (n = 8, rsd = 14%) of the matrix spike, which were all within the acceptable range of values.

The measured Hg concentrations in samples ranged from 0.04 to 5.6 ng in units of mass, which were almost all higher than the method detection limit (MDL) of 0.04 ng in units of mass (1.27 ng g^-1^). Two wood samples from sugar maple had measured values equal to the MDL.

### Statistical analysis

We treated the concentration of Hg measured for one composite sample of three trees as one individual observation in the following analyses. We log-transformed the data for all the analyses to meet the assumption of normality of the residuals.

To test the effects of tissue type on Hg concentrations, we applied a general linear model with tissue type as the main effect for each species in different sites. Because Hg concentration differed mainly by tissue type, we tested the effects of species and sites on Hg concentrations using a two-way ANOVA for each tissue type individually.

To express Hg content on a land-area basis, the average tissue concentration was multiplied by the estimated biomass of bark, foliage, branches, and bole wood for each species in hardwood and conifer stands at the four sites. Aboveground biomass in foliage, bark, branches, and wood was estimated using stand inventory specific to Huntington Forest [39], Sleepers River [33], Hubbard Brook [40] and Bear Brook [41] and allometric models developed for these species at Hubbard Brook [42]. To calculate the ratio of branch bark to wood, we used a weighted average of wood and bark based on the species and elements reported by Whittaker et al [43]. We estimated Hg content in branches using Hg concentrations of bark and wood times the estimated biomass in branch bark and wood. To estimate Hg concentrations of minor species that were not collected in this study, we used the average Hg concentration of dominant species. We summed across all the trees to obtain the total content of Hg in both hardwoods and conifer stands for each tissue type and site.

Statistical analyses were conducted with SAS 9.4 (SAS Institute Inc. 2013).

## Results

### Concentrations of Hg

Foliage, with means by species and site ranging from 11 to 48 ng g^-1^, had the highest Hg concentrations. Bole wood had the lowest concentrations (0.4–2.8 ng g^-1^), and bark was intermediate (4–26 ng g^-1^). This pattern was consistent across all the species and sites (*p* < 0.001 using least square means in a general linear model).

**Fig 2:**
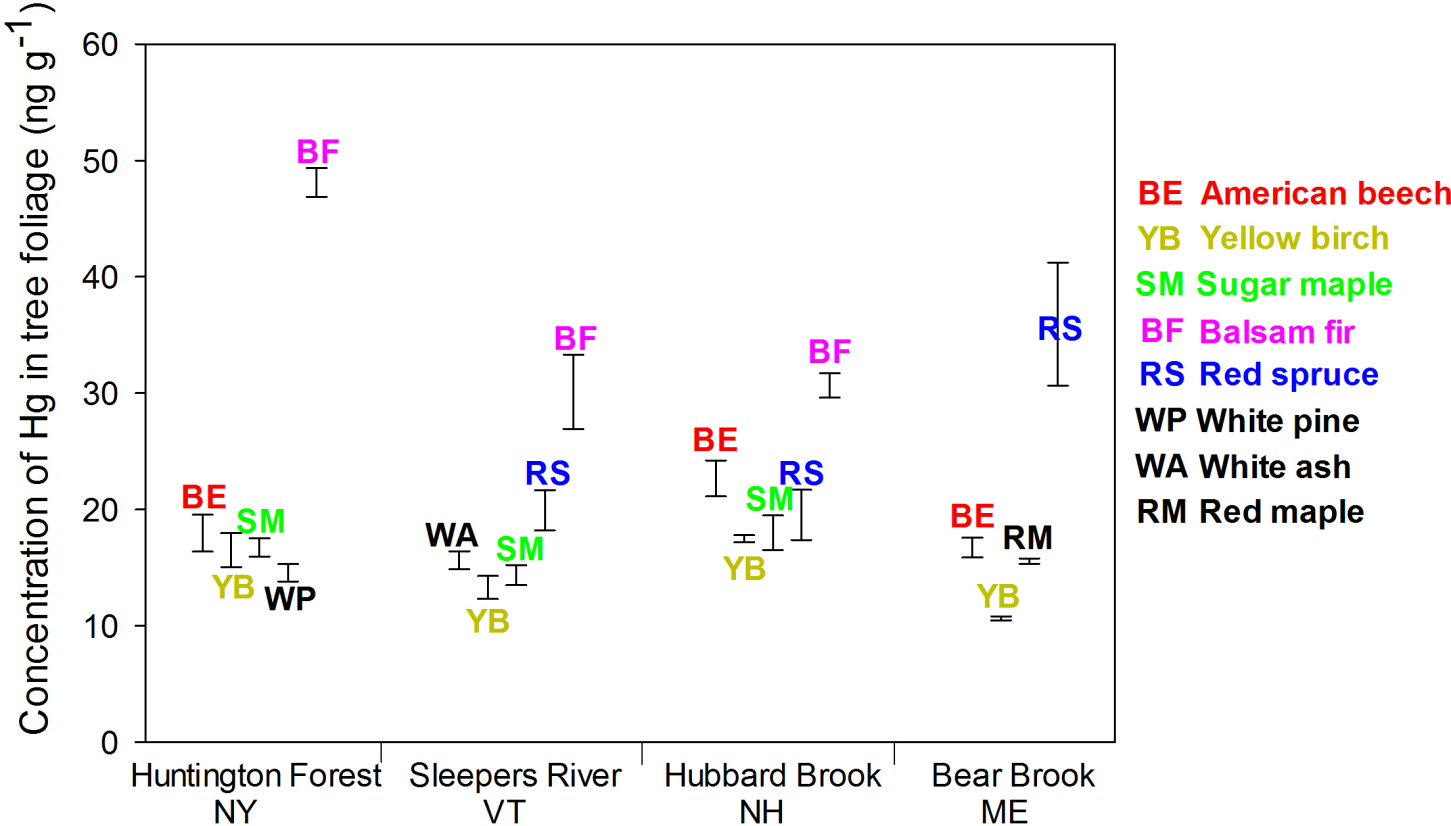
Concentrations of Hg in foliage of dominant species at four sites in the northeastern USA. Tree species included American beech (BE), yellow birch (YB), red maple (RM), sugar maple (SM), red spruce (RS), white ash (WA), white pine (WP) and balsam fir (BF). Error bars represent the SE of Hg concentrations of three composited samples.

**Fig 3:**
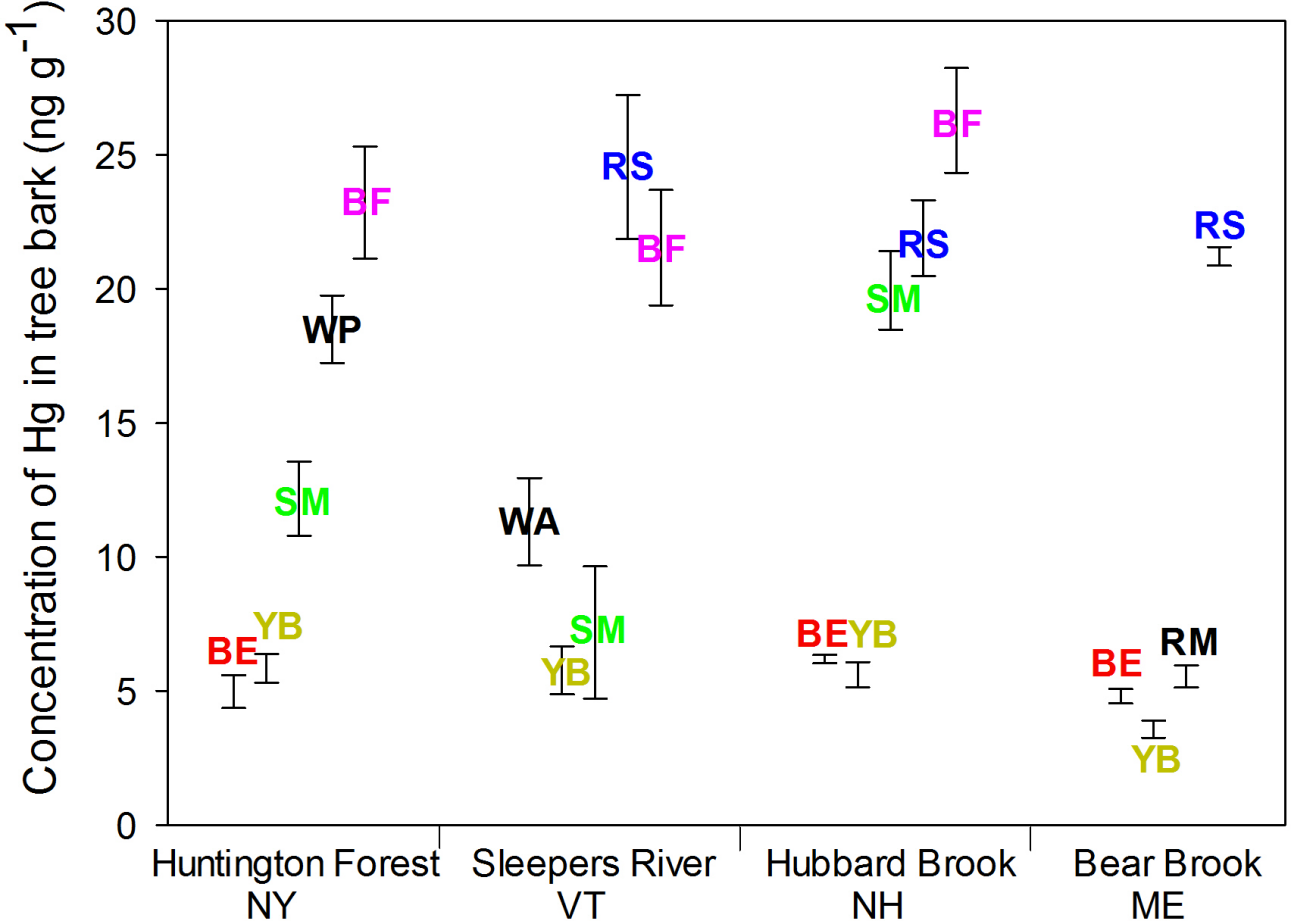
Concentrations of Hg in bark of dominant species at four sites in the northeastern USA. Tree species included American beech (BE), yellow birch (YB), red maple (RM), sugar maple (SM), red spruce (RS), white ash (WA), white pine (WP) and balsam fir (BF). Error bars represents the SE of Hg concentrations of three composited samples.

**Fig 4:**
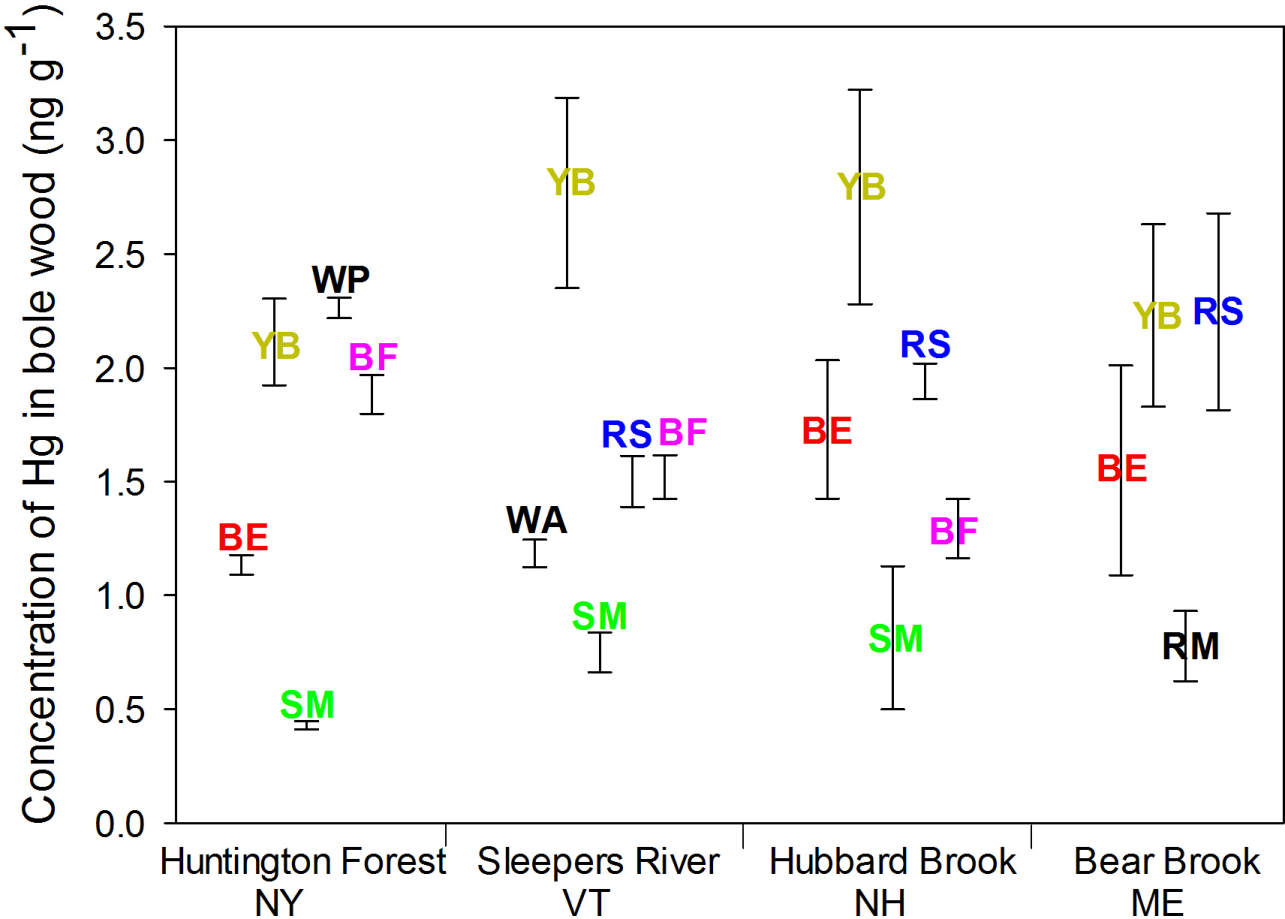
Concentrations of Hg in bole wood of dominant species at four sites in the northeastern USA. Tree species included American beech (BE), yellow birch (YB), red maple (RM), sugar maple (SM), red spruce (RS), white ash (WA), white pine (WP) and balsam fir (BF). Error bars represent the SE of Hg concentrations of three composited samples.

Species also differed in Hg concentrations. For foliage, concentrations of Hg were higher in balsam fir (30–48 ng g^-1^, depending on the site) and red spruce (20–37 ng g^-1^); the other species ranged from 10–23 ng g^-1^ (average for each species within site) (p < 0.001; Fig 2). For bark, likewise, concentrations of Hg in balsam fir (22–26 ng g^-1^) and red spruce (21–25 ng g^-1^) were higher than in the other species (4–20 ng g^-1^) (p < 0.001; Fig 3). For bole wood, concentrations of Hg were higher in yellow birch (2.1–2.8 ng g^-1^) and white pine (2.3 ng g^-1^) than in the other species (0.4–2.2 ng g^-1^) (*p* < 0.001; Fig 4).

Sites differed in concentrations of Hg for foliage (*p* < 0.001) and bark (*p* < 0.001) but not for bole wood (*p* = 0.24). For foliage, Hg concentrations were lower at Sleepers River (13–30 ng g^-1^ depending on the species) than Huntington Forest (15–48 ng g^-1^), Hubbard Brook (17–31 ng g^-1^), and Bear Brook (11–38 ng g^-1^). For bark, the concentration of Hg was lower at Bear Brook (4–21 ng g^-1^) than at Huntington Forest (6–26 ng g^-1^), Sleepers River (6–25 ng g^-1^) and Hubbard Brook (6–26 ng g^-1^). Concentrations of Hg in bole wood was similar among the four sites (Fig 4).

### Mercury pools

On a land-area basis, non-leaf tissues contained more Hg than did foliage in hardwood stands, because of their greater mass, in spite of lower Hg concentrations. The average biomass by tissue type in the hardwood stands was 111 Mg ha^-1^ for bole wood, 11 Mg ha^-1^ for bark and 3 Mg ha^-1^ for foliage. Across all four hardwood stands, bole wood Hg averaged 0.16 g ha^-1^ and bark averaged 0.10 g ha^-1^, while foliage averaged 0.06 g ha^-1^ (Table 2). For conifer stands, this pattern held only at Hubbard Brook. At Huntington Forest, Sleepers River and Bear Brook, contents of Hg in foliage (averaging 0.05 g ha^-1^ across three sites) were comparable to or larger than those in bole wood (0.03 g ha^-1^) and bark (0.04 g ha^-1^), because of the high Hg concentration of conifer foliage.

**Table 2:** Biomass and Hg content of foliage, bark and bole wood in hardwood and conifer stands in this study and three published studies.

Study location	Stand type^a^	Foliage		Bark		Bole wood		Branches		Reference
		Biomass (Mg ha^-1^)	Hg content (g ha^-1^)	Biomass (Mg ha^-1^)	Hg content (g ha^-1^)	Biomass (Mg ha^-1^)	Hg content (g ha^-1^)	Biomass (Mg ha^-1^)	Hg content (g ha^-1^)	
Huntington, NY, USA	Beech-maple	2.2	0.04	9.4	0.08	104.8	0.11	49.4	0.24	This study
	Fir-pine	1.7	0.05	2.5	0.05	20.7	0.04	8.6	0.11	
Sleepers River, VT, USA	Ash-maple	1.4	0.02	5.6	0.04	50.0	0.05	23.4	0.11	This study
	Spruce-fir	0.5	0.01	1.0	0.01	7.5	0.01	0.9	0.01	
Hubbard Brook, NH, USA	Beech-maple	7.6	0.15	21.4	0.26	221.7	0.36	53.0	0.37	This study
	Spruce-fir	4.7	0.11	8.7	0.12	73.3	0.14	1.1	0.02	
Bear Brook, ME, USA	Beech-maple	2.0	0.03	6.6	0.03	67.9	0.11	27.5	0.09	This study
	Spruce	2.3	0.09	2.9	0.06	21.9	0.05	0.9	0.12	
Dongling, Beijing, China	Chinese pine	13.5	0.43	5.8	0.02	51.7	0.14	24.5	0.48	*Zhou et al*., *2017*
	Oak	5.7	0.20	8.8	0.33	793.8	0.11	54.7	0.69	
	Larch	4.8	0.19	7.5	0.20	67.5	0.15	17.6	0.33	
	Birch-Carya	1.1	0.05	3.0	0.06	27.2	0.08	9.4	0.12	
New Hampshire and Vermont, USA	Beech-maple	5.7	0.18	N/A	N/A	24.5	0.15	N/A	N/A	*Richardson and Friedland*, *2015*
	Spruce-fir	1.6	0.15	N/A	N/A	9.1	0.30	N/A	N/A	
Washington, USA	Red Alder	2	0.03	N/A	N/A	113	< d.l.	N/A	N/A	*Obrist et al*., *2012*
	Douglas fir	3	0.32	N/A	N/A	136	0.54	N/A	N/A	

^a^ Oak refers to *Quercus liaotungensis* Mayr. Chinese pine refers to *Pinus tabulaeformis* Carr. Larch refers to *Larix principis-rupprechtii* Mayr. Birch refers to *Betula platyphylla* Suk and Carya refers to *Carya cathayensis* Sarg.

For the total aboveground Hg pool (with branch Hg concentrations estimated as intermediate between bark and wood), the hardwood stands contained more Hg than the conifer stands at Hubbard Brook (1.14 and 0.39 g ha^-1^), Sleepers River (0.22 and 0.04 g ha^-1^) and Huntington Forest (0.47 and 0.25 g ha^-1^), because of the greater biomass in hardwood stands. The estimated total aboveground biomass was 304 Mg ha^-1^ in the hardwood stand but only 88 Mg ha^-1^ in the conifer stand at Hubbard Brook, 80 compared to 10 Mg ha^-1^ at Sleepers River and 165 compared to 34 Mg ha^-1^ at Huntington Forest. At Bear Brook, where the conifer stand (28 Mg ha^-1^) was only modestly less massive than the hardwood stand (104 Mg ha^-1^), the conifer stand had a larger content of Hg (0.32 g ha^-1^) than the hardwood stand (0.26 g ha^-1^), due to the high concentration of Hg in conifer needles.

## Discussion

### Concentrations of Hg in foliage, bark and wood

Our finding of larger Hg concentrations in foliage (mean of 21 ng g^-1^ across species and site) and bark (mean of 13 ng g^-1^) than in wood (mean of 2 ng g^-1^) was consistent with studies in Norway [44], Ontario Canada [14, 23], Washington USA [27], and Vermont and New Hampshire USA [45]. This pattern was also documented in a review paper of Hg concentrations in forests [46] and a study of tree Hg concentrations across 14 sites in the USA [25]. Foliage has high Hg concentration because foliage absorbs Hg^0^ from the atmosphere through stomata [47], and Hg is accumulated over months for hardwood species [3, 12] and over years for conifers [48, 49]. Thus, it is not surprising that deciduous foliage collected late in the growing season and conifer needles including those older than one year had higher Hg concentrations than bark and wood. Foliage collected from hardwood species in early spring might have lower Hg concentrations than bark; at Huntington Forest, foliage collected on June 1^st^ from American beech, sugar maple and yellow birch had Hg concentrations of only 4 to 7 ng g^-1^, whereas foliage collected on August 1^st^ had concentrations four times higher [13].

Bark and wood have less exposure to atmospheric Hg than the foliage. Bark can capture and retain atmospheric Hg through surface sorption. A recent study of Australia pine (*Pinus nigra* J.F.Arnold) found that tree bark first absorb gaseous Hg^0^ or captured particulate Hg on the surface. Then, Hg was bound to thiol-containing molecules or tannins [50, 51]. Mercury recently deposited on the surface of leaves and bark was not included in our analysis, as we removed dust and other foreign material from the tissue surfaces before analysis. The fact that Hg concentrations in wood are so low suggests that little Hg moves from the foliage through the phloem [46], from bark into the wood [21, 52] or from roots to aboveground tissues in xylem sap [6].

### Effect of species and site on Hg concentrations

The observation that different tree species are characterized by different Hg concentrations in foliage is not surprising, because the main pathway of Hg from the atmosphere to foliage is through gas exchange via stomata. Species differ in rates of leaf gas exchange [53, 54], which might account for the variation in foliar Hg that we observed.

Concentration differences reported among tree species have not been consistent among studies. In this study, concentrations of Hg in balsam fir and red spruce foliage were twice those in the other species. This pattern of higher foliar Hg concentration in conifers than hardwood species agrees with studies in Ontario Canada [48], Washington USA [27], and Vermont and New Hampshire USA [45]. Higher foliar Hg concentrations in American beech than red spruce have been reported in New York [28] and Vermont and New Hampshire [45], probably because only needles up to 2 years old were sampled. We observed higher Hg concentrations in conifer needles, collecting needles of age classes up to 5 years old. In a survey of 45 sites in the Adirondacks, conifer needles in the 2-year age class had higher Hg concentrations than did hardwood leaves, but the current-year needles had lower Hg concentrations [28]. Other studies have also reported Hg accumulation in needles of older age classes in black pine (*Pinus nigra* J.F.Arnold) [55] and balsam fir and red spruce [56].

Mercury concentrations in bark and wood have less often been reported. Our observation that balsam fir and red spruce had higher Hg concentration in bark than other species might suggest that conifers have a higher rate of Hg sorption from the bark surface than hardwood species. The high Hg concentrations we observed in bole wood in yellow birch and white pine might be due to greater rates of Hg transport from either roots or foliage.

We found that sites differed in Hg concentrations in bark and foliage, but not in bole wood. Because foliage and bark are exposed to the atmosphere, Hg concentrations in these tissues may be more influenced by atmospheric Hg. The lower Hg concentration in bark at Bear Brook than in other three sites might be due to low Hg deposition in this site (Fig 1) due to its distance from Hg emission sources in the Midwest and urban centers of the northeastern U.S. [34, 35]. Lack of an effect of site on Hg concentrations in bole wood could be due to the limited transport of Hg to wood from the environment, as discussed above.

### Pools of Hg

The relative contribution of tree tissues to Hg pools at the study locations depends on both tissue concentrations and the biomass of those tissues, both of which vary across stands and sites (Table 2). The larger content of Hg in bole wood than in foliage in our hardwood stands was not surprising, because of the much larger biomass of bole wood than foliage. In this study, the bole wood biomass was 34–47 times foliar biomass, whereas the foliar Hg concentration was only 11–21 times bole wood Hg concentration across our four hardwood stands (Table 2). Similarly, wood contained more Hg than foliage for individual hardwood trees in Ontario, Canada [57] and for mixed hardwood forests in Beijing, China [58] and in New Brunswick, Canada [56]. Thus, wood can be at least as important as foliage for Hg budgets of forests. Note that our hardwood foliage samples were collected in late summer. The Hg concentration in foliage collected in October might be 50–70% higher, based on observations of beech, sugar maple and yellow birch in the Adirondacks [13]. If so, the Hg pool in foliage would then be 0.09 g ha^-1^, still less than the Hg pool in wood (0.16 g ha^-1^).

In contrast, the content of Hg in bole wood in conifer stands was not always larger than the needles at our four sites. Though the bole wood biomass was 8–12 times the biomass of needles, the Hg concentration in needles was 16–17 times the wood Hg concentration, depending on the site. Previous studies of conifers have reported greater Hg content of bole wood than needles: a Douglas-fir stand in Washington State, the bole wood was reported to contain more Hg (0.5 g ha^-1^) than the needles (0.3 g ha^-1^), and in eight spruce-fir stands in New Hampshire and Vermont, the bole wood contained more Hg (0.3 g ha^-1^) than the needles (0.2 g ha^-1^) [45]. However, in both of these studies, only recent age classes of needles were collected (up to two years), which might underestimate Hg concentrations by a factor of 2, based on observations of balsam fir and red spruce in New Brunswick, Canada [56]. It is probably not uncommon for conifer forests to have more Hg in needles than in wood, due to the long exposure of evergreen foliage to the atmosphere, while for deciduous forests, foliage is a smaller Hg pool than bole wood.

### Relationship to previous Hg studies in the same stands

In a study of throughfall, litter inputs, and gaseous emissions of elemental mercury from the soil surface at the Huntington Forest, New York, Hg inputs were higher and losses were lower under conifers compared to hardwoods [17]. Our results appear consistent with this difference, as conifers seem to accumulate Hg to a greater degree than hardwoods. However, the amount of Hg contained in trees is too small to explain the budget discrepancy of 0.1 g ha^-1^ yr^-1^ [17]. Soil Hg pools in northern forests (~300 g ha^-1^) are orders of magnitude greater than those in trees [17, 36], so accumulation in soils is a more likely mechanism to explain the discrepancy in Hg budgets between stands at this site.

At Huntington Forest, the foliage of the same three hardwood species we sampled in 2015 was sampled on a monthly basis throughout the growing season in 2009 and 2010 [17]. Using their reported daily Hg accumulation rate [17], we estimated foliar Hg concentrations for the exact leaf age we sampled (early August) in 2009 and 2010. Foliar Hg concentrations appear to be decreasing over time, from 21.3 ± 0.8 ng g^-1^ in 2009 and 19.7 ± 0.3 ng g^-1^ in 2010, to 17.0 ± 0.4 ng g^-1^ in 2015. The rate of decrease is thus -0.7 ± 0.1% yr^-1^ (*p* = 0.002), using a simple linear regression with time as the independent variable and treating the three species as replicates. Similarly, fluxes of Hg in leaf litter Hg declined from 2004 to 2015 at Huntington Forest [59]. Concentrations of atmospheric Hg^0^ at Huntington Forest were reported to decline with a slope of -1.6 ± 2.0% yr^-1^ from 2005 to 2008 and a slope of -1.0 ± 2.0% yr^-1^ from 2009 to 2014 associated with declines in regional emissions [60]. Thus, the decreases in foliar Hg likely reflect the decline in atmospheric Hg, the major source of Hg in tree foliage. Measurements of Hg concentration in foliage or litterfall would appear to be an effective approach to monitor future changes in atmospheric Hg deposition. Note that a biomonitoring program involving conifer species would need to specify needle ages since Hg concentration in needles vary by age class [55].

## Conclusions

Concentrations of Hg were studied in tree foliage, bark and bole wood for eight species in hardwood and conifer stands in four study sites in the northeastern USA “S1 Table”. Concentrations of Hg varied by tree tissue type, with foliage > bark > bole wood in both hardwood and conifer stands. Concentrations of Hg in foliage and bark varied significantly with species and site, with species the more important explanatory variable. Concentrations of Hg in bole wood differed by species but not by site, suggesting that translocation of Hg from foliage, roots and bark is limited.

The Hg content of aboveground biomass varied by forest type and site. Bole wood contained more Hg than foliage and bark in hardwood stands because of the much larger biomass of bole wood; conifer stands had larger Hg concentrations in foliage than in wood because of the higher concentrations in conifer needles than deciduous leaves. Understanding the distribution of Hg in tree tissues could help inform management of Hg pools and fluxes and policy decisions regarding the fate of harvested biomass.

## Supporting information

S1 Table Concentrations of Hg in each composited tissue sample in this study. (DOCX)
