# Supplementary material for: Concentrations and content of mercury in bark, wood, and leaves in hardwoods and conifers in four forested sites in the northeastern USA
Source: PLoS One. 2018 Apr 23;13(4):e0196293. doi: 10.1371/journal.pone.0196293 (PMC5912732; doi:10.1371/journal.pone.0196293)
Supplement: S1 Table — (DOCX) [file pone.0196293.s001.docx]

**Supplementary Information**

**Table 1.** Concentrations of Hg in each Composited Tissue Sample in this Study.

| **Site** | **Stand type** | **Tree species^a^** | **Tissue type** | **Composited replicates** | **Concentrations of Hg (ng g^-1^)** |
| --- | --- | --- | --- | --- | --- |
| bear brook | hardwood | BE | bark | 1 | 4.79 |
| bear brook | hardwood | BE | bark | 2 | 5.28 |
| bear brook | hardwood | BE | bark | 3 | 4.34 |
| bear brook | hardwood | BE | leave | 1 | 16.95 |
| bear brook | hardwood | BE | leave | 2 | 18.05 |
| bear brook | hardwood | BE | leave | 3 | 15.12 |
| bear brook | hardwood | BE | wood | 1 | 2.47 |
| bear brook | hardwood | BE | wood | 2 | 1.14 |
| bear brook | hardwood | BE | wood | 3 | 1.04 |
| bear brook | hardwood | RM | bark | 1 | 5.14 |
| bear brook | hardwood | RM | bark | 2 | 6.37 |
| bear brook | hardwood | RM | bark | 3 | 5.12 |
| bear brook | hardwood | RM | leave | 1 | 15.86 |
| bear brook | hardwood | RM | leave | 2 | 15.13 |
| bear brook | hardwood | RM | leave | 3 | 15.68 |
| bear brook | hardwood | RM | wood | 1 | 0.64 |
| bear brook | hardwood | RM | wood | 2 | 0.60 |
| bear brook | hardwood | RM | wood | 3 | 1.08 |
| bear brook | conifer | RS | bark | 1 | 21.21 |
| bear brook | conifer | RS | bark | 2 | 20.62 |
| bear brook | conifer | RS | bark | 3 | 21.82 |
| bear brook | conifer | RS | leave | 1 | 44.51 |
| bear brook | conifer | RS | leave | 2 | 31.32 |
| bear brook | conifer | RS | leave | 3 | 37.04 |
| bear brook | conifer | RS | wood | 1 | 3.07 |
| bear brook | conifer | RS | wood | 2 | 2.06 |
| bear brook | conifer | RS | wood | 3 | 1.61 |
| bear brook | hardwood | YB | bark | 1 | 4.20 |
| bear brook | hardwood | YB | bark | 2 | 3.24 |
| bear brook | hardwood | YB | bark | 3 | 3.28 |
| bear brook | hardwood | YB | leave | 1 | 10.89 |
| bear brook | hardwood | YB | leave | 2 | 10.72 |
| bear brook | hardwood | YB | leave | 3 | 10.30 |
| bear brook | hardwood | YB | wood | 1 | 2.42 |
| bear brook | hardwood | YB | wood | 2 | 2.81 |
| bear brook | hardwood | YB | wood | 3 | 1.46 |
| hubbard brook | hardwood | BE | bark | 1 | 6.30 |
| hubbard brook | hardwood | BE | bark | 2 | 5.88 |
| hubbard brook | hardwood | BE | bark | 3 | 6.37 |
| hubbard brook | hardwood | BE | leave | 1 | 25.72 |
| hubbard brook | hardwood | BE | leave | 2 | 20.66 |
| hubbard brook | hardwood | BE | leave | 3 | 21.62 |
| hubbard brook | hardwood | BE | wood | 1 | 1.22 |
| hubbard brook | hardwood | BE | wood | 2 | 2.27 |
| hubbard brook | hardwood | BE | wood | 3 | 1.70 |
| hubbard brook | conifer | BF | bark | 1 | 22.38 |
| hubbard brook | conifer | BF | bark | 2 | 28.39 |
| hubbard brook | conifer | BF | bark | 3 | 28.08 |
| hubbard brook | conifer | BF | leave | 1 | 28.75 |
| hubbard brook | conifer | BF | leave | 2 | 32.35 |
| hubbard brook | conifer | BF | leave | 3 | 30.89 |
| hubbard brook | conifer | BF | wood | 1 | 1.55 |
| hubbard brook | conifer | BF | wood | 2 | 1.14 |
| hubbard brook | conifer | BF | wood | 3 | 1.19 |
| hubbard brook | conifer | RS | bark | 1 | 24.68 |
| hubbard brook | conifer | RS | bark | 2 | 21.00 |
| hubbard brook | conifer | RS | bark | 3 | 20.02 |
| hubbard brook | conifer | RS | leave | 1 | 17.29 |
| hubbard brook | conifer | RS | leave | 2 | 17.44 |
| hubbard brook | conifer | RS | leave | 3 | 23.83 |
| hubbard brook | conifer | RS | wood | 1 | 2.07 |
| hubbard brook | conifer | RS | wood | 2 | 1.96 |
| hubbard brook | conifer | RS | wood | 3 | 1.80 |
| hubbard brook | hardwood | SM | bark | 1 | 21.23 |
| hubbard brook | hardwood | SM | bark | 2 | 17.03 |
| hubbard brook | hardwood | SM | bark | 3 | 21.58 |
| hubbard brook | hardwood | SM | leave | 1 | 16.51 |
| hubbard brook | hardwood | SM | leave | 2 | 16.48 |
| hubbard brook | hardwood | SM | leave | 3 | 20.94 |
| hubbard brook | hardwood | SM | wood | 1 | 1.43 |
| hubbard brook | hardwood | SM | wood | 2 | 0.41 |
| hubbard brook | hardwood | SM | wood | 3 | 0.60 |
| hubbard brook | hardwood | YB | bark | 1 | 6.04 |
| hubbard brook | hardwood | YB | bark | 2 | 6.11 |
| hubbard brook | hardwood | YB | bark | 3 | 4.67 |
| hubbard brook | hardwood | YB | leave | 1 | 17.54 |
| hubbard brook | hardwood | YB | leave | 2 | 16.92 |
| hubbard brook | hardwood | YB | leave | 3 | 17.95 |
| hubbard brook | hardwood | YB | wood | 1 | 1.89 |
| hubbard brook | hardwood | YB | wood | 2 | 2.84 |
| hubbard brook | hardwood | YB | wood | 3 | 3.52 |
| huntington forest | hardwood | BE | bark | 1 | 3.77 |
| huntington forest | hardwood | BE | bark | 2 | 5.76 |
| huntington forest | hardwood | BE | bark | 3 | 5.40 |
| huntington forest | hardwood | BE | leave | 1 | 19.26 |
| huntington forest | hardwood | BE | leave | 2 | 14.81 |
| huntington forest | hardwood | BE | leave | 3 | 19.78 |
| huntington forest | hardwood | BE | wood | 1 | 1.15 |
| huntington forest | hardwood | BE | wood | 2 | 1.05 |
| huntington forest | hardwood | BE | wood | 3 | 1.20 |
| huntington forest | conifer | BF | bark | 1 | 19.25 |
| huntington forest | conifer | BF | bark | 2 | 24.07 |
| huntington forest | conifer | BF | bark | 3 | 26.33 |
| huntington forest | conifer | BF | leave | 1 | 47.18 |
| huntington forest | conifer | BF | leave | 2 | 46.57 |
| huntington forest | conifer | BF | leave | 3 | 50.61 |
| huntington forest | conifer | BF | wood | 1 | 1.74 |
| huntington forest | conifer | BF | wood | 2 | 2.04 |
| huntington forest | conifer | BF | wood | 3 | 1.87 |
| huntington forest | hardwood | SM | bark | 1 | 14.94 |
| huntington forest | hardwood | SM | bark | 2 | 10.62 |
| huntington forest | hardwood | SM | bark | 3 | 10.97 |
| huntington forest | hardwood | SM | leave | 1 | 16.73 |
| huntington forest | hardwood | SM | leave | 2 | 18.07 |
| huntington forest | hardwood | SM | leave | 3 | 15.35 |
| huntington forest | hardwood | SM | wood | 1 | 0.41 |
| huntington forest | hardwood | SM | wood | 2 | 0.41 |
| huntington forest | hardwood | SM | wood | 3 | 0.47 |
| huntington forest | conifer | WP | bark | 1 | 16.10 |
| huntington forest | conifer | WP | bark | 2 | 20.36 |
| huntington forest | conifer | WP | bark | 3 | 19.04 |
| huntington forest | conifer | WP | leave | 1 | 13.09 |
| huntington forest | conifer | WP | leave | 2 | 15.45 |
| huntington forest | conifer | WP | leave | 3 | 15.10 |
| huntington forest | conifer | WP | wood | 1 | 2.23 |
| huntington forest | conifer | WP | wood | 2 | 2.21 |
| huntington forest | conifer | WP | wood | 3 | 2.35 |
| huntington forest | hardwood | YB | bark | 1 | 5.87 |
| huntington forest | hardwood | YB | bark | 2 | 6.75 |
| huntington forest | hardwood | YB | bark | 3 | 4.90 |
| huntington forest | hardwood | YB | leave | 1 | 16.22 |
| huntington forest | hardwood | YB | leave | 2 | 19.14 |
| huntington forest | hardwood | YB | leave | 3 | 14.07 |
| huntington forest | hardwood | YB | wood | 1 | 2.45 |
| huntington forest | hardwood | YB | wood | 2 | 2.09 |
| huntington forest | hardwood | YB | wood | 3 | 1.80 |
| sleepers river | hardwood | WA | bark | 1 | 8.97 |
| sleepers river | hardwood | WA | bark | 2 | 14.44 |
| sleepers river | hardwood | WA | bark | 3 | 10.54 |
| sleepers river | hardwood | WA | leave | 1 | 14.78 |
| sleepers river | hardwood | WA | leave | 2 | 14.93 |
| sleepers river | hardwood | WA | leave | 3 | 17.17 |
| sleepers river | hardwood | WA | wood | 1 | 1.25 |
| sleepers river | hardwood | WA | wood | 2 | 1.24 |
| sleepers river | hardwood | WA | wood | 3 | 1.06 |
| sleepers river | conifer | BF | bark | 1 | 19.30 |
| sleepers river | conifer | BF | bark | 2 | 19.47 |
| sleepers river | conifer | BF | bark | 3 | 25.83 |
| sleepers river | conifer | BF | leave | 1 | 28.71 |
| sleepers river | conifer | BF | leave | 2 | 25.44 |
| sleepers river | conifer | BF | leave | 3 | 36.21 |
| sleepers river | conifer | BF | wood | 1 | 1.51 |
| sleepers river | conifer | BF | wood | 2 | 1.69 |
| sleepers river | conifer | BF | wood | 3 | 1.36 |
| sleepers river | conifer | RS | bark | 1 | 19.63 |
| sleepers river | conifer | RS | bark | 2 | 25.17 |
| sleepers river | conifer | RS | bark | 3 | 28.86 |
| sleepers river | conifer | RS | leave | 1 | 18.26 |
| sleepers river | conifer | RS | leave | 2 | 23.39 |
| sleepers river | conifer | RS | leave | 3 | 18.12 |
| sleepers river | conifer | RS | wood | 1 | 1.43 |
| sleepers river | conifer | RS | wood | 2 | 1.35 |
| sleepers river | conifer | RS | wood | 3 | 1.72 |
| sleepers river | hardwood | SM | bark | 1 | 4.16 |
| sleepers river | hardwood | SM | bark | 2 | 5.31 |
| sleepers river | hardwood | SM | bark | 3 | 11.05 |
| sleepers river | hardwood | SM | leave | 1 | 15.71 |
| sleepers river | hardwood | SM | leave | 2 | 14.62 |
| sleepers river | hardwood | SM | leave | 3 | 12.73 |
| sleepers river | hardwood | SM | wood | 1 | 0.92 |
| sleepers river | hardwood | SM | wood | 2 | 0.66 |
| sleepers river | hardwood | SM | wood | 3 | 0.67 |
| sleepers river | hardwood | YB | bark | 1 | 7.55 |
| sleepers river | hardwood | YB | bark | 2 | 4.93 |
| sleepers river | hardwood | YB | bark | 3 | 4.81 |
| sleepers river | hardwood | YB | leave | 1 | 15.17 |
| sleepers river | hardwood | YB | leave | 2 | 11.88 |
| sleepers river | hardwood | YB | leave | 3 | 12.81 |
| sleepers river | hardwood | YB | wood | 1 | 3.22 |
| sleepers river | hardwood | YB | wood | 2 | 3.15 |
| sleepers river | hardwood | YB | wood | 3 | 1.93 |

^a^ tree species included American beech (BE), yellow birch (YB), red maple (RM), sugar maple (SM), red spruce (RS), white ash (WA), white pine (WP) and balsam fir (BF).
